# Novel inflammation-combined prognostic index to predict survival outcomes in patients with gastric cancer

**DOI:** 10.18632/oncotarget.28353

**Published:** 2023-01-31

**Authors:** Noriyuki Hirahara, Takeshi Matsubara, Shunsuke Kaji, Hikota Hayashi, Yohei Sasaki, Koki Kawakami, Ryoji Hyakudomi, Tetsu Yamamoto, Yoshitsugu Tajima

**Affiliations:** ^1^Department of Digestive and General Surgery, Shimane University Faculty of Medicine, Izumo, Shimane 693-8501, Japan; ^2^Department of Surgery, Matsue Red Cross Hospital, Matsue, Shimane 690-0886, Japan; ^3^Department of Surgery, Masuda Red Cross Hospital, Masuda, Shimane 698-8501, Japan

**Keywords:** gastric cancer, laparoscopic gastrectomy, novel predictive index, inflammation-combined prognostic index, cancer-specific survival

## Abstract

Background: We focused on the lymphocyte-to-monocyte ratio (LMR), neutrophil-to-lymphocyte ratio (NLR), and platelet-to-lymphocyte ratio (PLR) and devised an inflammation-combined prognostic index (ICPI) as a prognostic marker of cancer-specific survival (CSS).

Methods: We reviewed the clinicopathological data of 480 patients with gastric cancer undergoing curative laparoscopic gastrectomy between 2009 and 2019. This study examined the significance of LMR, NLR, PLR, and ICPI as cancer-specific prognostic markers.

Results: In univariate analysis, tumor diameter, histological differentiation, pathological tumor-node-metastasis (pTNM) stage, LMR, NLR, PLR, C-reactive protein (CRP) level, carcinoembryonic antigen (CEA), and postoperative chemotherapy were significantly associated with CSS. In multivariate analysis, pTNM stage and CEA were the independent risk factors for CSS, although LMR, NLR, and PLR were not the independent risk factors for CSS.

The ICPI formula was constructed using hazard ratios for three inflammation-based biomarkers with worse prognosis identified in the univariate analysis: LMR <4.315, NLR ≥2.344, and PLR ≥212.01, which were each scored as 1, with all remaining values pointed at 0. ICPI was calculated as follows: ICPI = 2.9 × LMR + 2.8 × NLR + 2.8 × PLR. The optimal cutoff value of ICPII was 2.9. On multivariate analysis, pTNM stage, CEA, and ICPI were independent prognostic factors for CSS. In the Kaplan–Meier survival analysis, CSS in the high ICPI group was significantly worse than that in the low ICPI group.

Conclusion: ICPI was devised as a novel predictive index for prognosis, and its usefulness was clarified.

## INTRODUCTION

Tumor-related systemic inflammation based on host–tumor interaction between cancer loci and individuals is caused not only by local nutritional malabsorption but also by systemic metabolic disorders [1, 2]. Systemic inflammation must be evaluated objectively and dynamically as it changes constantly during multidisciplinary treatment. Against this background, the usefulness of biomarkers has been attracting attention in recent years, and it is desirable to devise biomarkers that can evaluate these dynamic changes more quickly, easily, accurately, and at a lower cost [3, 4]. Biomarkers are generally classified into three categories in cancer treatment: (1) having diagnostic significance for cancer, (2) serving as prognostic indicators, and (3) predicting therapeutic effects or risk of side effects. Although it may not be possible to develop significant markers that predict all three categories, tumor-related systemic inflammation and metabolic malnutrition in patients with cancer occur not only in advanced cancers but also in relatively early-stage cancers and are prognostic factors independent of pathological factors and induce treatment resistance [5, 6]. Therefore, it is reasonable to include systemic inflammation and metabolic nutritional status as indicators when devising biomarkers. If the prognosis can be predicted using the pre-treatment specimens, it will lead to the identification of a group of patients who require multimodal treatment, including aggressive chemotherapy and radiotherapy, which will lead to individualized treatment and improved prognosis [6, 7]. Occasionally, even stage 1 cases may recur after surgery. In this study, we focused on blood cell components that complementarily reflect systemic inflammation and metabolic status and devised a cancer-specific prognostic marker for all patients, not limited to patients in stages II and III.

In this study, we focused on blood cell components that complementarily reflect systemic inflammation and metabolic status and devised a cancer-specific prognostic marker.

## RESULTS

### Association between the inflammatory biomarkers and clinicopathological features

The 480 patients were divided into the low and high groups based on the cutoff values of each inflammatory biomarker (Table 1); 207 patients (43.1% [male, 154; female, 53]) showed low LMR (median age, 74 [range, 38–91] years). Moreover, 273 patients (56.9% [male, 183; female, 90]) showed high LMR (median age, 69 [range, 36–89] years). Furthermore, 296 (61.7% [male, 204; female, 92]) patients showed low NLR (median age, 70 [range, 36–91] years), and 184 (38.3% [male, 133; female, 51]) patients showed high NLR (median age, 74 [range, 43–90] years). A total of 407 (84.8% [male, 286; female, 121]) patients showed low PLR (median age, 70 [range, 36–90] years), and 73 (15.2% [male, 51; female, 22]) showed high PLR (median age, 72 [range, 43–91] years).

**Table 1:** Association between the inflammatory biomarkers and clinicopathological features

Characteristics	Total patients	LMR			NLR			PLR		
		<4.315 (*n* = 207)	≥4.315 (*n* = 273)	*p* value	<2.344 (*n* = 296)	≥2.344 (*n* = 184)	*p* value	<212.01 (*n* = 407)	≥212.01 (*n* = 73)	*p* value
Age (years)		74 (38–91)	69 (36–89)	<0.001	70 (36–91)	74 (43–90)	0.008	70 (36–90)	72 (43–91)	0.062
Sex				0.079			0.432			0.944
Male	337	154	183		204	133		286	51	
Female	143	53	90		92	51		121	22	
ASA–PS				<0.001			0.002			0.007
1	25	5	20		19	6		22	3	
2	409	171	238		259	150		354	55	
3	46	31	15		18	28		31	15	
BMI		21.9 (14.0–32.5)	22.8 (14.8–40.4)	<0.001	22.3 (14.7–40.4)	22.3 (14.0–32.7)	0.754	22.4 (14.7–40.4)	21.7 (14.0–32.5)	0.063
WBC		5730 (2870–13700)	5630 (510–9830)	0.292	5460 (510–9280)	6115 (3510–13700)	<0.001	5710 (510–13700)	5460 (1830–12730)	0.133
Neutrophil		3700 (1310–11460)	3190 (250–6910)	<0.001	3010 (250–5100)	4270 (2210–11460)	<0.001	3340 (250–8494)	3850 (1100–11460)	0.012
Lymphocyte		1310 (230–3780)	1850 (230–3780)	<0.001	1845 (230–3780)	1255 (230–2270)	<0.001	1730 (230–3780)	960 (230–2020)	<0.001
Monocyte		408 (210–937)	311 (3–727)	<0.001	339 (3–937)	366 (85–829)	<0.001	348 (3–937)	362 (37–829)	0.192
Platelet		216 (58–726)	222 (39–460)	0.432	220 (39–460)	220 (58–726)	0.267	215 (39–460)	283 (119–726)	<0.001
Tumor location				0.891			0.842			0.84
EGJ	15	7	8		8	7		13	2	
U	93	43	50		58	35		76	17	
M	204	85	119		129	75		174	30	
L	168	72	96		101	67		144	24	
Tumor diameter (mm)		44 (3–176)	40 (4–180)	0.01	40 (3–180)	42 (5–176)	0.059	40 (3–180)	50 (16–150)	0.001
Differentiation				0.657			0.44			0.431
Well	94	37	57		63	31		83	11	
Moderate	177	76	101		109	68		146	31	
Poor	209	94	115		124	85		178	31	
Depth of tumor				0.003			0.01			<0.001
T1a-1b	252	89	163		171	81		226	26	
2	62	29	33		39	23		55	7	
3	71	38	33		39	32		59	12	
4a-4b	95	51	44		47	48		67	28	
Lymph node meta				0.004			0.332			0.011
N0	314	123	191		203	111		278	36	
N1	57	29	28		32	25		41	16	
N2	56	32	24		31	25		46	10	
N3	53	23	30		30	23		42	11	
pTNM stage				<0.001			0.004			<0.001
1a-1b	283	99	184		192	91		255	28	
2a-2b	87	50	37		47	40		67	20	
3a-3c	110	58	52		57	53		85	25	
Operative procedure				0.001			0.209			0.034
Total	101	58	43		58	43		78	23	
Proximal	50	14	36		36	14		46	4	
Distal	329	135	194		202	127		283	46	
Operation time (min)		390 (204–911)	378 (70–808)	0.51	381 (158–911)	386 (70–703)	0.5	383 (70–911)	386 (231–692)	0.288
Intraope. blood loss		50 (0–3600)	20 (0–5850)	0.03	40 (0–5850)	50 (0–2600)	0.568	40 (0–5850)	30 (0–1600)	0.921
Postoperative complications				0.036			0.486			0.419
Present	145	73	72		86	59		120	25	
Absent	335	134	201		210	125		287	48	
CRP (mg/dl)		0.11 (0.01–11.10)	0.06 (0.01–4.26)	<0.001	0.07 (0.01–5.35)	0.11 (0.01–11.1)	<0.001	0.07 (0.01–11.1)	0.16 (0.01–7.09)	<0.001
CEA (ng/ml)		3.4 (0.7–171.6)	3.3 (0.7–86.4)	0.23	3.2 (0.7–106.0)	3.6 (0.7–171.6)	0.064	3.3 (0.7–171.6)	3.4 (0.8–163.3)	0.566
Adjuvant chemotherapy				0.919			0.558			0.557
Yes	131	56	75		78	53		109	22	
No	349	151	198		218	131		298	51	

### Cox regression analysis of inflammatory biomarkers associated with cancer-specific survival (CSS)

In univariate analysis, tumor diameter (*p* < 0.001), histological differentiation (*p* = 0.029), pathological TNM (pTNM) stage (*p* < 0.001), LMR (hazard ratio [HR], 2.866; *p* < 0.001), NLR (HR, 2.778; *p* < 0.001), PLR (HR, 2.803; *p* = 0.001), C-reactive protein (CRP) level (*p* = 0.003), carcinoembryonic antigen (CEA) (*p* = 0.011), and postoperative chemotherapy (*p* < 0.001) were significantly associated with CSS. In multivariate analysis, pTNM stage (HR, 21.452; 95% confidence interval [CI], 2.268–8.151; *p* < 0.001) and CEA (HR, 2.000; 95% CI, 1.089–3.672; *p* = 0.025) were identified as independent risk factors for CSS, although LMR, NLR, and PLR were not found to be independent risk factors for CSS (Table 2).

**Table 2:** Cox regression analysis of inflammatory biomarkers associated with cancer-specific survival

Variables	Category or characteristics	Patients (*n* = 480)	Univariate			Multivariate		
			HR	95% CI	*p* value	HR	95% CI	*p* value
Age	(<70/≥70)	225/255	1.373	1.134–1.662	0.258			
Sex	(female/male)	143/337	1.534	0.782–3.009	0.196			
BMI	(≥18.5/<18.5)	439/41	1.013	0.364–2.824	0.980			
Tumor diameter	(<5/≥5)	285/195	4.299	2.268–8.151	<0.001	1.713	0.875–3.354	0.116
Differentiation	(well and mod/poor)	271/209	1.895	1.067–3.364	0.029	0.822	0.447–1.511	0.528
pTNM stage	(1/2,3)	283 /197	40.385	9.799–166.437	<0.001	21.452	4.549–101.176	<0.001
LMR	(≥4.315/<4.315)	273/207	2.866	1.585–5.181	<0.001	1.523	0.760–3.074	0.234
NLR	(<2.344/≥2.344)	296/184	2.778	1.557–4.956	<0.001	1.563	0.782–3.123	0.206
PLR	(<212.069/≥212.069)	407/73	2.803	1.503–5.227	0.001	1.170	0.570–2.403	0.669
CRP	(≦0.5/>0.5)	413/67	2.664	1.409–5.037	0.003	0.954	0.472–1.929	0.896
CEA	(<5.0/≥5.0)	364/116	2.210	1.231–3.966	0.011	2.000	1.089–3.672	0.025
Postope. Complications	(absent/present)	335/145	1.280	0.695–2.358	0.428			
Adjuvant chemotherapy	(no/yes)	349/131	6.206	3.329–11.570	<0.001	1.371	0.675–2.787	0.383

### CSS according to inflammatory biomarkers

**Figure 1:**
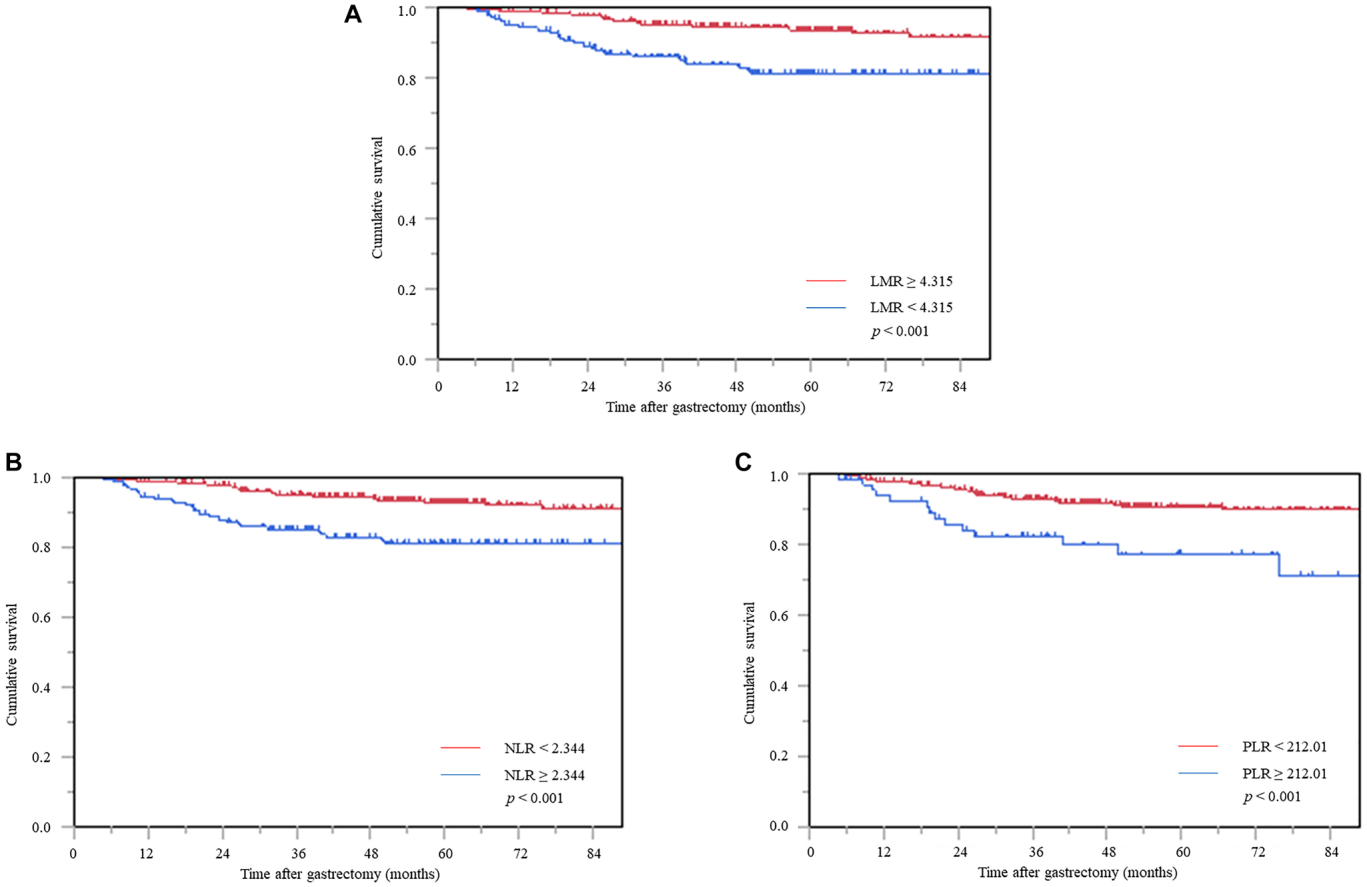
Cancer-specific survival curve based on the inflammatory biomarkers. (**A**) Lymphocyte-to-monocyte ratio, (**B**) neutrophil-to-lymphocyte ratio, and (**C**) platelet-to-lymphocyte ratio. Abbreviations: LMR: Lymphocyte-to-monocyte ratio, NLR: Neutrophil-to-lymphocyte ratio; PLR: Platelet-to-lymphocyte ratio.

The Kaplan–Meier survival curve revealed significantly worse CSS in the low LMR (*p* < 0.001) (Figure 1A), high NLR (*p* < 0.001) (Figure 1B), and high PLR (*p* < 0.001) groups (Figure 1C).

### Inflammation-based prognostic index formula

The inflammation-based prognostic index (ICPI) formula was constructed using HRs for three inflammation-based biomarkers with worse prognosis identified in the univariate analysis. LMR <4.315, NLR ≥2.344, and PLR ≥212.01, which were each scored as 1, with all remaining values pointed at 0.

The ICPI was calculated as follows: ICPI = 2.9 × LMR + 2.8 × NLR + 2.8 × PLR.

Using ROC analysis, the optimal cutoff value of ICPII was 2.9 based on CSS (sensitivity, 0.715%; specificity, 0.583%; area under the curve [AUC], 0.656).

### Association between ICPI and clinicopathological features

A total of 329 (68.5% [male, 226; female, 103]) patients showed low ICPI (median age, 74 [range, 38–91] years), and 151 (31.5% [male, 111; female, 40]) patients showed high ICPI. The ICPI was significantly correlated with age, American Society of Anaesthesiologists Physical Status, white blood cell count, neutrophil count, lymphocyte count, monocyte count, platelet count, tumor diameter, tumor depth, lymph node metastasis, pTNM stage, operative procedure, and CRP (Table 3).

**Table 3:** Relationships between the inflammation-combined prognostic index and clinicopathological features

Characteristics	Total patients	ICPI		*p* value
		≤2.9	>2.9	
		(*n* = 329)	(*n* = 151)	
Age (years)		70 (36–90)	74 (43–91)	0.001
Sex				0.281
Male	337	226	111	
Female	143	103	40	
ASA-PS				<0.001
1	25	21	4	
2	409	288	121	
3	46	20	26	
BMI		22.4 (14.7–40.4)	22.0 (14.0–32.5)	0.102
WBC		5580 (510–9830)	5910 (2880–13700)	0.001
Neutrophil		3110 (250–6910)	4070 (1310–11460)	<0.001
Lymphocyte		1820 (230–3780)	1190 (230–2100)	<0.001
Monocyte		332 (3–937)	382 (165–829)	<0.001
Platelet		218 (39–460)	230 (58–726)	0.029
Tumor location				0.592
EGJ	15	8	7	
U	93	62	31	
M	204	143	61	
L	168	116	52	
Tumor diameter (mm)		40 (3–180)	45 (5–176)	0.013
Differentiation				0.644
Well	94	68	26	
Moderate	177	121	56	
Poor	209	140	69	
Depth of tumor				<0.001
T1a-1b	252	189	63	
2	62	46	16	
3	71	43	28	
4a-4b	95	51	44	
Lymph node meta				0.044
N0	314	229	85	
N1	57	34	23	
N2	56	33	23	
N3	53	33	20	
pTNM stage				<0.001
1a-1b	283	216	67	
2a-2b	87	50	37	
3a-3c	110	63	47	
Operative procedure				0.044
Total	101	60	41	
Proximal	50	39	11	
Distal	329	230	99	
Operation time (min)		380 (70–911)	386 (204–703)	0.641
Intraoperative blood loss		40 (0–5850)	50 (0–2600)	0.314
Postoperative complications				0.252
Present	145	94	51	
Absent	335	235	100	
CRP (mg/dl)		0.06 (0.01–5.35)	0.12 (0.01–11.10)	<0.001
CEA (ng/ml)		3.3 (0.7–106.0)	3.4 (0.7–171.6)	0.134
Adjuvant chemotherapy				0.54
Yes	131	87	44	
No	349	242	107	

### Comparison of predictive ability of inflammatory biomarkers for CSS

**Figure 2:**
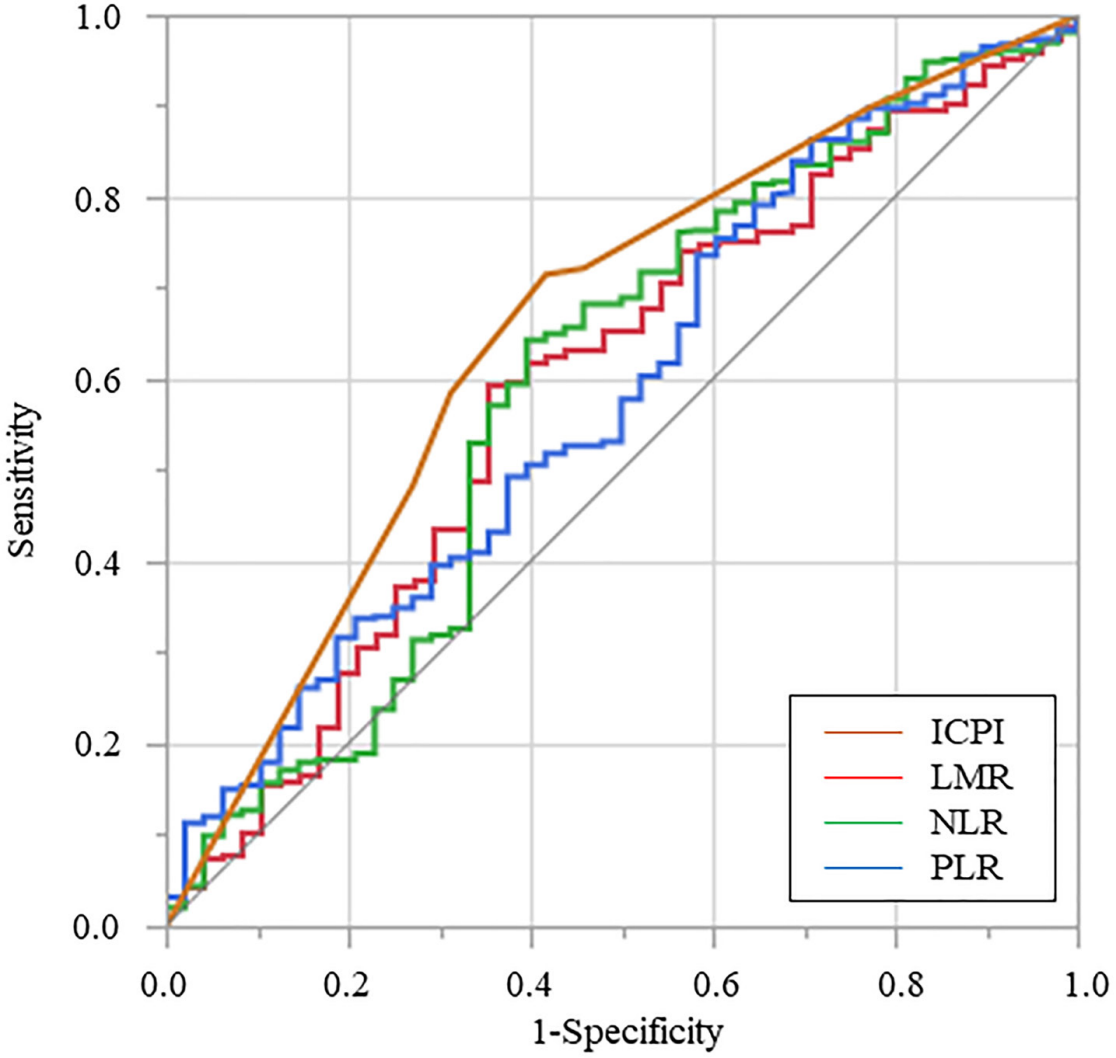
Receiver operating characteristic curve for cancer-specific survival was plotted to verify the optimum cutoff value of lymphocyte-to-monocyte ratio, neutrophil-to-lymphocyte ratio, platelet-to-lymphocyte ratio, and inflammation-based prognostic index. Abbreviations: LMR: Lymphocyte-to-monocyte ratio, NLR: Neutrophil-to-lymphocyte ratio; PLR: Platelet-to-lymphocyte ratio, ICPI: Inflammation-combined prognostic index.

The AUC estimate method was used to compare the predictive ability of the inflammatory biomarkers. The AUCs of LMR, NLR, PLR, and ICPI were 0.594, 0.596, 0.585, and 0.656, respectively. The AUCs of ICPI were significantly higher than those of LMR (*p* = 0.029), NLR (*p* = 0.018), and PLR (*p* = 0.005) (Figure 2).

### Cox regression analysis of ICPI associated with CSS

On multivariate analysis, pTNM stage (HR, 22.646; 95% CI, 4.826–106.277; *p* < 0.001), CEA (HR, 2.050; 95% CI, 1.120–2.570; *p* = 0.020), and ICPI (HR, 2.511; 95% CI, 1.383–4.562; *p* = 0.003) were confirmed as independent prognostic factors for CSS (Table 4).

**Table 4:** Cox regression analysis of the inflammation-combined prognostic index associated with cancer-specific survival

Variables	Category or characteristics	Patients (*n* = 480)	Univariate			Multivariate		
			HR	95% CI	*p* value	HR	95% CI	*p* value
Age	(<70/≥70)	225/265	1.373	1.134–1.662	0.258			
Sex	(female/male)	143/337	1.534	0.782–3.009	0.196			
BMI	(≥18.5/<18.5)	439/41	1.013	0.364–2.824	0.980			
Tumor diameter	(<5/≥5)	285/195	4.299	2.268–8.151	<0.001	1.656	0.851–3.221	0.138
Differentiation	(well and mod/poor)	271/209	1.895	1.067–3.364	0.029	0.798	0.439–1.450	0.460
pTNM stage	(1/2,3)	283/197	40.385	9.799–166.437	<0.001	22.646	4.826–106.277	<0.001
ICPI	(≦2.9/>2.9)	329/151	3.757	2.115–6.674	<0.001	2.511	1.383–4.562	0.003
CRP	(≦0.5/>0.5)	413/67	2.664	1.409–5.037	0.003	0.987	0.496–1.964	0.971
CEA	(<5.0/≥5.0)	364/116	2.210	1.231–3.966	0.011	2.050	1.120–3.753	0.020
Postope. Complication	(absent/present)	335/145	1.280	0.695–2.358	0.428			
Adjuvant chemotherapy	(no/yes)	349/131	6.206	3.329–11.570	<0.001	1.281	0.639–2.570	0.485

### CSS according to ICPI

**Figure 3:**
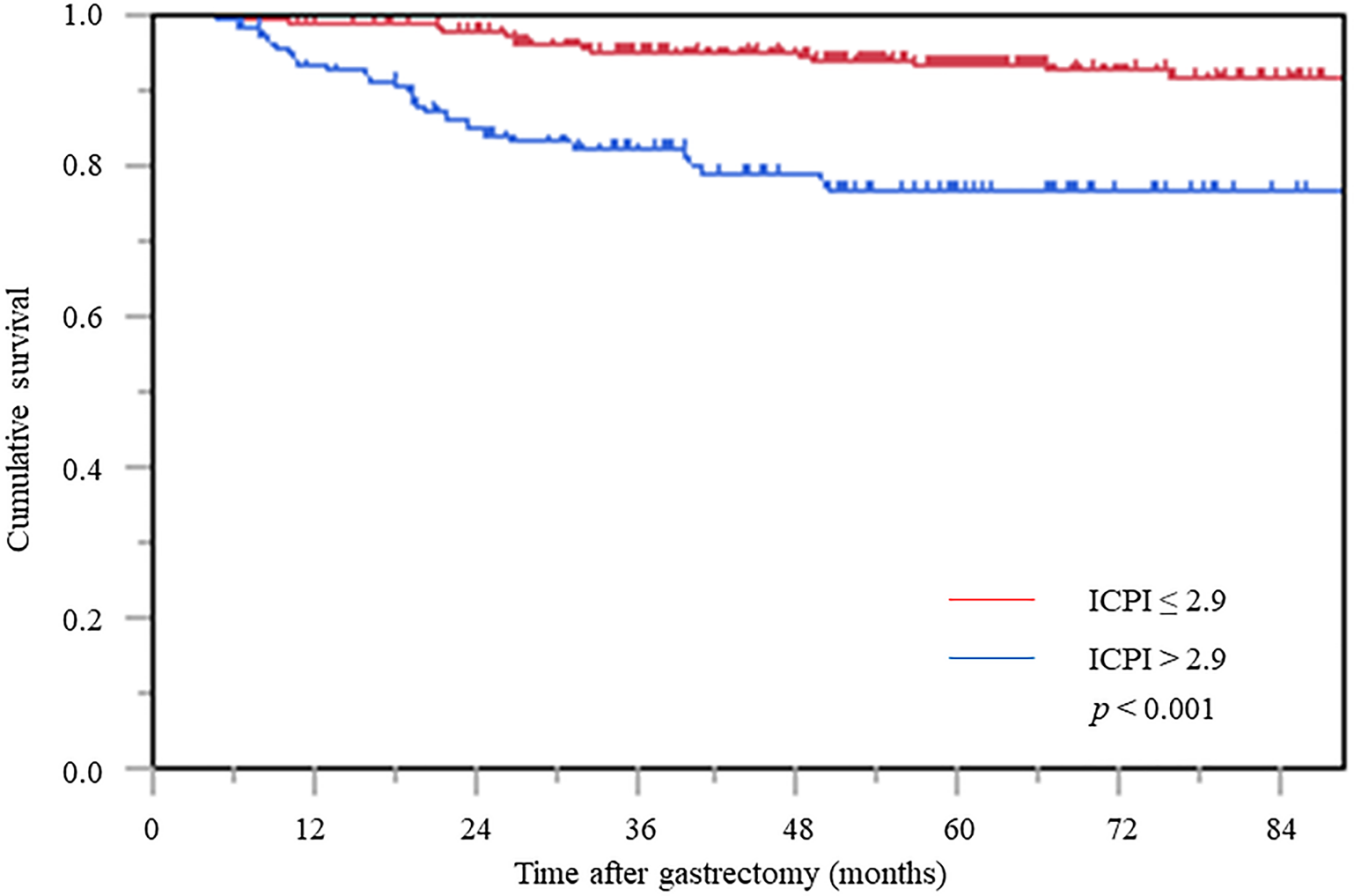
Cancer-specific survival curve based on the inflammation-based prognostic index. Abbreviation: ICPI: inflammation based prognostic index.

In the Kaplan–Meier survival analysis, CSS in the high ICPI group was significantly worse than that in the low ICPI group (Figure 3).

**Figure 4:**
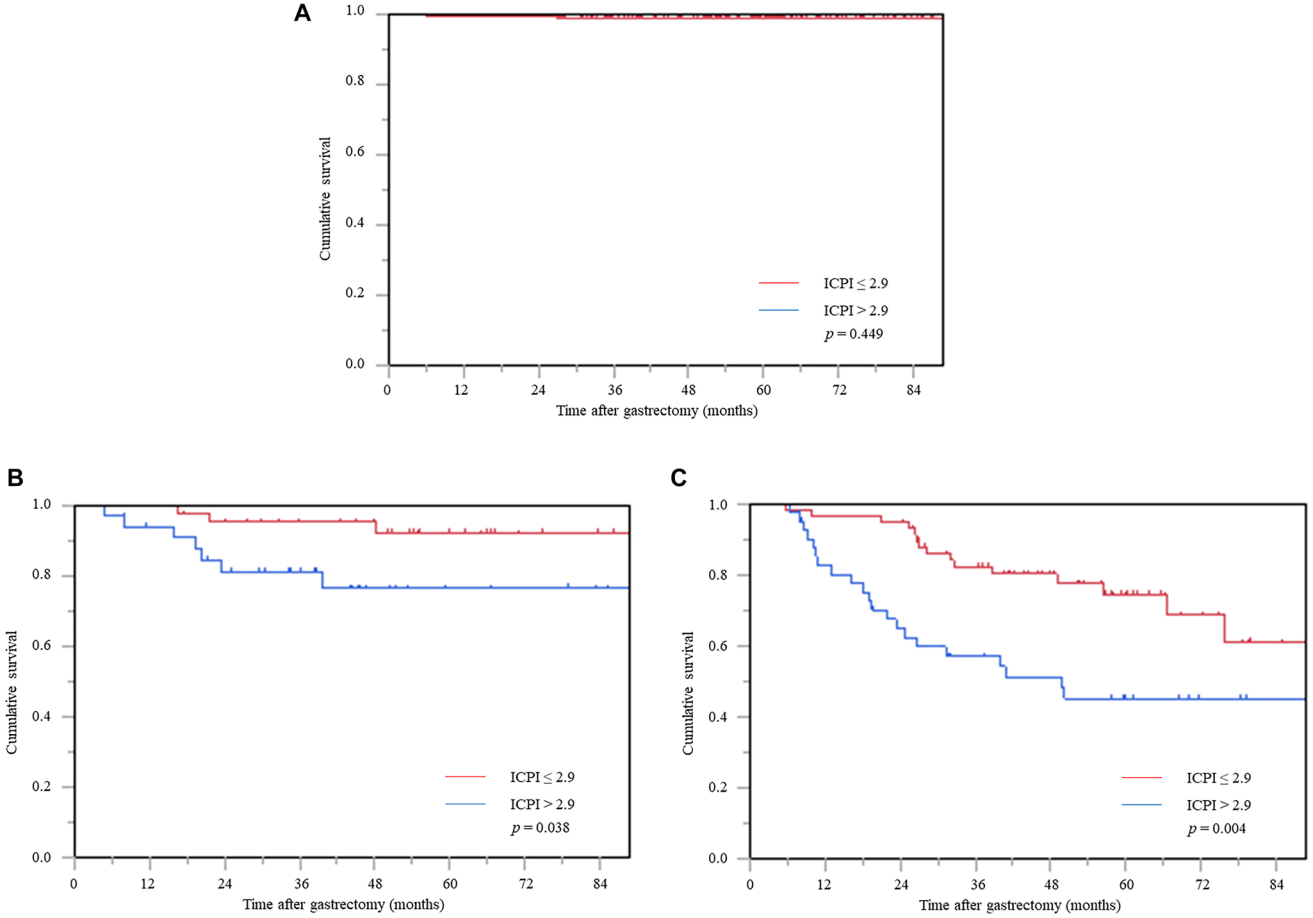
Cancer-specific survival curve based on the inflammation-based prognostic index in the stage stratification analysis. (**A**) Stage I, (**B**) stage II, and (**C**) stage III. Abbreviation: ICPI: inflammation based prognostic index.

Furthermore, in stage stratification analysis, the high ICPI group was significantly associated with worse prognosis in stages II and III, whereas the prognosis of stage I patients did not reach statistical significance among the ICPI values (Figure 4A–4C).

## DISCUSSION

*In vivo* inflammatory responses are involved in cancer growth, invasion, and metastasis, and the involvement of systemic inflammatory responses and the surrounding microenvironment is intricately intertwined [5, 8, 9]. Tumor necrosis factor-α (TNF-α), granulocyte colony-stimulating factor, interleukin-1 (IL-1), and IL-6 are produced by tumor cells and can induce a tumor-related systemic inflammatory reaction (SIR) [10, 11]. Among them, IL-6 is a multifunctional inflammatory cytokine that causes the proliferation and differentiation of various types of cells, such as immunocompetent and hematopoietic cells [12, 13]. Thus, dynamic changes in SIR resulting from tumor–host interaction can be accurately assessed by direct measurement of cytokines. However, routine measurement of cytokines in patients with cancer in clinical practice is expensive and impractical. In contrast, LMR, NLR, and PLR assessments using neutrophils, lymphocytes, monocytes, and platelets, which are regulated by cytokines, proliferate, and differentiate, are simple methods to evaluate the systemic inflammatory response using blood cell components and are complementary to each other [14, 15].

Neutrophils regulate the tumor microenvironment by producing pro-inflammatory cytokines/chemokines that promote proliferation, invasion, and metastasis of cancer cells, such as matrix metalloproteinase-9 and anti-apoptotic factor (nuclear factor kappa light chain enhancer of activated B cells) [16]. Furthermore, increased neutrophils produce large amounts of nitric oxide, arginase, and reactive oxygen species, which not only impair T-cell activation and reduce extracellular matrix adhesion but also promote angiogenesis and cellular DNA damage and inhibit tumor cell apoptosis [17]. As a result, a favorable microenvironment for tumor cells is established, which promotes tumor growth and metastasis. Lymphocytes function as an important component of the immune complex and serve as an antitumor immune response by inducing cytotoxic cell death and inhibiting tumor cell proliferation and migration. In addition, lymphocytes secrete cytokines, such as interferon-γ and TNF-α, which regulate cancer cell growth and metastasis through cellular and humoral immune mechanisms [18, 19]. It has also been shown to be a useful marker for screening nutritional status. Monocytes in the peripheral blood migrate to tissues, mature, and differentiate into macrophages. In patients with cancer, macrophages infiltrating the stroma of tumor tissues are called tumor-associated macrophages, which suppress tumor immunity and promote cancer cell proliferation by releasing angiogenic factors and inhibiting cytotoxic T cells [20]. Platelets allow circulating tumor cells to escape host immune surveillance via platelet-derived transforming growth factor-β and direct platelet-tumor cell contact to induce epithelial-mesenchymal transition, angiogenesis, and differentiation of cancer-associated fibroblasts and regulatory T cells. As a result, it induces microvascular permeability, which promotes the extravasation of cancer cells and induces distant metastasis [21].

Thus, LMR expresses the immune response in the tumor microenvironment and is an indicator of individual immunity [22, 23]. PLR serves as a marker for the balance between the inflammatory reaction and immune response of the host [24, 25]. NLR was initially reported as a predictor of outcome in critically ill patients admitted to intensive care units, but it has since been reported as an oncological prognostic marker and is the most evidence-accumulating biomarker [26, 27]. Since these biomarkers reflect different pathological conditions in patients with cancer, it is necessary to integrate and evaluate the three biomarkers to predict the prognosis of cancer more accurately. In this study, each inflammatory marker showed significant differences in univariate analysis but was not extracted as an independent prognostic factor in the multivariate analysis. Considering that these inflammatory markers calculated from two types of blood cell components are insufficient as prognostic predictors, we devised a novel biomarker reflecting systemic inflammation.

Since the HR is a numerical value that objectively compares the relative risk, we devised the ICPI, which is a novel prognostic marker calculated by adding the specific gravity provided to the prognosis of each inflammatory marker using the HR in univariate analysis. As a result, it was proven that the AUC value of ICPI was significantly higher than that of each inflammatory marker, demonstrating its high predictive and diagnostic ability. Furthermore, ICPI could be extracted as an independent prognostic factor in multivariate analysis.

We have previously reported the usefulness of an index calculated by adding the number of markers that recognized a significant difference in esophageal cancer, ignoring the specific gravity provided to the prognosis of each inflammatory marker. However, by considering the prognostic significance of each marker, the detection power of the index as a prognostic indicator increased.

Although ICPI is a new prognostic prediction index for cancer, sufficient attention is required for its interpretation. First, the number of cases was relatively small and included cases with a short postoperative follow-up period. Furthermore, some medicines, such as anticoagulants and anti-inflammatory agents, have not been evaluated. It is also necessary to measure inflammatory cytokines associated with tumors, and it is a future issue whether ICPI can be a prognostic index for other carcinomas. Second, we did not histologically examine leukocyte migration and infiltration into the cancer site. Third, the calculation formula is complicated, which impedes the generalization of this marker. Because the inflammatory biomarkers have similar hazard ratios, the ICBI formula may be simplified by unifying the coefficients to 2.8 or 2.9. Alternatively, removing the coefficient and adding the number of risk factor inflammation biomarkers will simplify the formula, but further examination is required in the future. In addition, further usefulness may be found by examining its association with the recurrence pattern.

In this study, the ICPI was devised as a novel predictive index of prognosis, and its usefulness was clarified. However, it is still unclear how active preoperative intervention using the ICPI as an indicator will contribute to improved oncological prognosis. In the future, it will be necessary to conduct a multicenter prospective study to examine the prognostic effect of preoperative interventions, including nutrition.

## MATERIALS AND METHODS

### Patients

We conducted a retrospective study of patients with gastric cancer who underwent curative laparoscopic gastrectomy between January 2009 and December 2019 at our institution. The average follow-up period for survival was 1743.3 days, and the median follow-up period was 1709 days (interquartile range, 969–2304). Clinical patients’ clinicopathological data and laboratory records were collected using an electronic medical records platform. Blood biochemical examination was performed within 1 week prior to the surgery. All patients were eligible for laparoscopic surgery, but we excluded patients with severe adhesion in the abdominal cavity. Furthermore, laparoscopic surgery was not indicated for patients in whom gastrectomy could not be performed without grasping the cancer site with forceps.

Gastrectomy and lymphadenectomy were usually performed according to the guidelines of the Japanese Gastric Cancer Association [28]. The postoperative stage was based on the 7th edition of the tumor-node-metastasis (TNM) system [29]. The severity of postoperative complications was graded according to the Clavien–Dindo (CD) classification [30]. CD grade II or higher complications were defined as the occurrence of any complications. Postoperative adjuvant chemotherapy with tegafur/gimeracil/oteracil potassium (S-1) was recommended for patients with stage II or higher gastric cancer, usually for 1 year. Furthermore, 5-fluorouracil-based chemotherapy regimens (cisplatin plus S-1 or capecitabine) were recommended to the majority of patients with recurrent gastric cancer according to the Japanese Gastric Cancer Treatment Guidelines (Version 4) [28].

### Inflammatory biomarkers

The lymphocyte-to-monocyte ratio (LMR) was calculated by dividing the absolute peripheral lymphocyte count by the absolute monocyte count, neutrophil-to-lymphocyte ratio (NLR) was calculated by dividing the absolute peripheral neutrophil count by the absolute lymphocyte count, and platelet-to-lymphocyte ratio (PLR) was calculated by dividing the absolute platelet count by the lymphocyte count.

The optimal cutoff values of the LMR, NLR, and PLR were determined via the receiver operating curve (ROC) analysis. The optimal cutoff values of LMR, NLR, and PLR for predicting cancer-specific survival (CSS) were 4.315, 2.344, and 212.1, respectively.

### Statistical analyses

CSS was defined as the date of gastrectomy until death due to gastric cancer.

Student’s *t*-test was used when assessing continuous variables, and the chi-squared test or Fisher’s test was used when assessing categorical variables. The survival rate was calculated using Kaplan–Meier analysis, and statistical analysis was performed using the log-rank test. Significantly associated variables (*p* < 0.05) in univariate analysis were included in the multivariate analysis using the Cox proportional hazards model to identify the independent factors. Probability values less than 0.05 were defined as statistically significant factors. Statistical analyses were performed using JMP software (version 16.0; SAS Institute, Cary, NC, USA).

## Abbreviations

AUC: area under the curve
CEA: carcinoembryonic antigen
CD: Clavien–Dindo
CI: confidence interval
CRP: C-reactive protein
CSS: cancer-specific survival
HR: hazard ratio
ICPI: inflammation-combined prognostic index
IL: interleukin
NLR: neutrophil-to-lymphocyte ratio
LMR: lymphocyte-to-monocyte ratio
PLR: platelet-to-lymphocyte ratio
pTNM: pathological tumor-node-metastasis
ROC: receiver operating curve
SIR: systemic inflammatory reaction
TNF-α: tumor necrosis factor-α
